# *Aedes aegypti* Molecular Responses to Zika Virus: Modulation of Infection by the Toll and Jak/Stat Immune Pathways and Virus Host Factors

**DOI:** 10.3389/fmicb.2017.02050

**Published:** 2017-10-23

**Authors:** Yesseinia I. Angleró-Rodríguez, Hannah J. MacLeod, Seokyoung Kang, Jenny S. Carlson, Natapong Jupatanakul, George Dimopoulos

**Affiliations:** W. Harry Feinstone Department of Molecular Microbiology and Immunology, Bloomberg School of Public Health, Johns Hopkins University, Baltimore, MD, United States

**Keywords:** Zika virus, dengue virus, *Aedes aegypti*, innate immunity, vector competence

## Abstract

Zika (ZIKV) and dengue virus (DENV) are transmitted to humans by *Aedes* mosquitoes. However, the molecular interactions between the vector and ZIKV remain largely unexplored. In this work, we further investigated the tropism of ZIKV in two different *Aedes aegypti* strains and show that the virus infection kinetics, tissue migration, and susceptibility to infection differ between mosquito strains. We also compare the vector transcriptome changes upon ZIKV or DENV infection demonstrating that 40% of the mosquito’s midgut infection-responsive transcriptome is virus-specific at 7 days after virus ingestion. Regulated genes included key factors of the mosquito’s anti-viral immunity. Comparison of the ZIKV and DENV infection-responsive transcriptome data to those available for yellow fever virus and West Nile virus identified 26 genes likely to play key roles in virus infection of *Aedes* mosquitoes. Through reverse genetic analyses, we show that the Toll and the Jak/Stat innate immune pathways mediate increased resistance to ZIKV infection, and the conserved DENV host factors vATPase and inosine-5′-monophosphate dehydrogenase are also utilized for ZIKV infection.

## Introduction

*Aedes* mosquitoes are the primary vectors of Zika virus (ZIKV), dengue virus (DENV), and chikungunya virus (CHIKV) which are currently the most devastating arboviral pathogens. DENV causes 400 million infections and 12,500 deaths per year, and ZIKV recently emerged as another arbovirus of major public health concern (Guzman et al., 2010; WHO, 2012; Bhatt et al., 2013; Weaver et al., 2016). Typically, ZIKV infections are mild or asymptomatic; however, in contrast to DENV and CHIKV infections, ZIKV has been associated with Guillain-Barré syndrome in adults, and a heightened risk of microcephaly and other birth defects in prenatally infected infants (Cao-Lormeau et al., 2016; Johansson et al., 2016). At the moment, no preventive or therapeutic drugs exist against these pathogens. Hence, the control of *Aedes* mosquitoes remains the primary approach to limit viral transmission (Morrison et al., 2008).

Currently, the primary DENV and ZIKV control approaches either suppress mosquito populations or render the vector less competent to support viruses infection (Harris et al., 2011; Walker et al., 2011; Carvalho et al., 2015). The most commonly used approach is based on insecticides, but insecticide resistance is becoming increasingly common and controlling spread of arboviruses will require novel approaches. New alternative strategies for DENV and ZIKV control are being developed, and include genetically modified mosquitoes that could block virus transmission, transmission-blocking vaccines and small molecules (Harris et al., 2011; Carvalho et al., 2015; Schmidt et al., 2017). Research in the past few decades on DENV-*Aedes* molecular interactions has been critical to the development of these new vector control approaches. However, the recent public health emergency of the CHIKV and ZIKV epidemics, and the ongoing global threat of yellow fever has emphasized the importance of broadening our understanding to other virus infection systems. A first step toward this goal is to understand how mosquitoes respond to, and manage, infection at the molecular level. Our more extensive knowledge of *A. aegypti* – DENV interactions can greatly leverage and facilitate this effort for other mosquito-arbovirus infection systems.

The mosquito becomes infected with an arbovirus when a female acquires a blood meal on an infected human. The ingested virus first encounters the midgut tissue, where it replicates to produce viral particles. The viral particles then disseminate from the midgut through the hemolymph to other tissues including the salivary glands. There they are further propagated before being transmitted to a new host during a subsequent blood meal. The average extrinsic incubation period (EIP) of both DENV and ZIKV is 7–14 days. The DENV EIP is dependent on numerous factors such as mosquito and virus genotypes, as well as environmental factors such as humidity and temperature (Hardy et al., 1983; Bennett et al., 2002; Black et al., 2002; Salazar et al., 2007; McElroy et al., 2008; Dubrulle et al., 2009; Tchankouo-Nguetcheu et al., 2010; Li et al., 2012; Chouin-Carneiro et al., 2016). Different mosquito populations and strains can vary in permissiveness to virus infection. This variability is driven by the virus’ compatibility with host factors and its ability to elicit and evade the action of the mosquito’s restriction factors, many of which are components of the insect’s innate immune system.

The immune responses of arthropods are, to a significant degree, regulated by the Toll, immune deficiency (Imd), and Janus kinase/signal transducer and activator of transcription (Jak/Stat) signaling pathways. Activation of these pathways leads to the nuclear translocation of transcription factors, resulting in the production of a variety of anti-pathogen effector molecules (Xi et al., 2008; Souza-Neto et al., 2009; Ramirez and Dimopoulos, 2010; Zou et al., 2011; Garver et al., 2013; Clayton et al., 2014). Furthermore, the RNAi pathway, a key antiviral defense system, can degrade viral RNAs. Functional genomics and reverse genetic approaches have demonstrated that the Toll, Jak/Stat, and RNAi pathways exert anti-DENV activity (Sanders et al., 2005; Campbell et al., 2008; Xi et al., 2008; Sánchez-Vargas et al., 2009; Souza-Neto et al., 2009; Ramirez and Dimopoulos, 2010; Sim et al., 2013). However, while the molecular interactions between *A. aegypti* and DENV have been characterized in some detail over the past decade, the interactions between ZIKV and its mosquito vector have remained largely unexplored.

Here we have initiated the elucidation of *A. aegypti* molecular response to ZIKV infection, and the implication of the mosquito’s immune system, and known virus host factors, in modulating vector competence. We demonstrate that different *A. aegypti* strains show different degrees of permissiveness to ZIKV and DENV. We used an RNA sequencing (RNAseq)-based comparative transcriptome analysis of *A. aegypti* responses to ZIKV and DENV infection of the midgut tissue that revealed both conserved and unique responses to infection with the two viruses, involving a variety of functional gene groups, including immunity. We show that the Toll and Jak/Stat pathways are implicated in suppressing ZIKV infection. We also report that key host factors for viral replication are essential for infection with both viruses. Furthermore, our comparative analysis of the ZIKV, DENV, West Nile virus (WNV) and yellow fever virus (YFV) infection-responsive transcriptomes identify potential pan-flavivirus infection-responsive genes that could represent key factors in the general mosquito-virus interactions. This study has significantly furthered our understanding of mosquito-virus interactions with a special emphasis on the largely understudied ZIKV.

## Results

### *Aedes aegypti* Strains Show Differential Susceptibility to ZIKV

**FIGURE 1:**
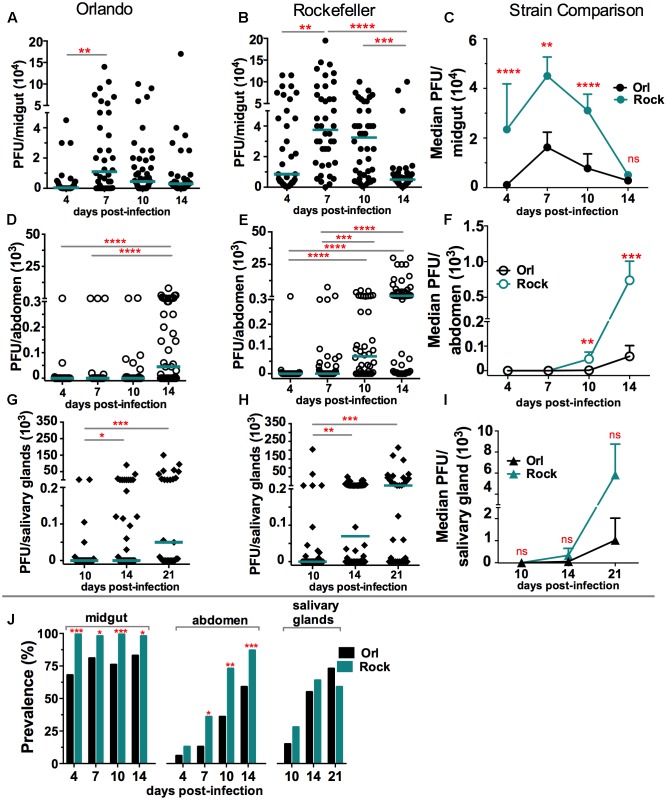
Zika virus (ZIKV) tropism in *A. aegypti*. Orlando (Orl) and Rockefeller (Rock) *A. aegypti* mosquito strains were infected with ZIKV via an infectious-blood meal, and **(A–C)** midguts, **(D–F)** abdomens, and **(G–I)** salivary glands were assessed for infection intensity at different time points. **(A)** 4 days (*N* = 34), 7 days (*N* = 36), 10 days (*N* = 46), 14 days (*N* = 36). **(B)** 4 days (*N* = 37), 7 days (*N* = 40), 10 days (*N* = 42), 14 days (*N* = 42). **(D)** 4 days (*N* = 35), 7 days (*N* = 38), 10 days (*N* = 25), 14 days (*N* = 51). **(E)** 4 days (*N* = 56), 7 days (*N* = 61), 10 days (*N* = 40), 14 days (*N* = 55). **(G)** 10 days (*N* = 33), 14 days (*N* = 38), 21 days (*N* = 27). **(H)** 10 days (*N* = 40), 14 days (*N* = 46), 21 days (*N* = 34). Each dot represents the plaque-forming units (PFUs) per individual tissue from three independent experiments. Bars represents the median. ^∗^*P* < 0.05, ^∗∗^*P* < 0.01, ^∗∗∗^*P* < 0.001; Kruskal-Wallis test. **(C,F,I)** show strain comparisons between Orl and Rock; each point represents the median PFU per strain at the given time point per replicate (*N* = 3). ^∗∗^*P* < 0.01, ^∗∗∗^*P* < 0.001, ^∗∗∗∗^*P* < 0.0001; Mann–Whitney test. **(J)** The prevalence (percentage) of infected mosquitoes for Orl vs. Rock was compared at each time point. ^∗^*P* < 0.05, ^∗∗^*P* < 0.01, ^∗∗∗^*P* < 0.001; chi square test.

Genetic variations among *A. aegypti* mosquito populations and strains can influence their susceptibility to different viruses. Here we assessed the possible differences in temporal and spatial (tissue) ZIKV tropism between two *A. aegypti* strains. These strains differ in their permissiveness to DENV at the stage of midgut and salivary gland infection; the Rockefeller (Rock) strain being the more permissive to DENV compared to the Orlando (Orl) strain (Sim et al., 2013). We orally infected mosquitoes by allowing them to feed on blood containing equal titers of ZIKV. Next, we assayed infection intensity and prevalence (**Figures 1A–J**), by plaque assay, at different days post-infectious blood meal (dpibm) in the midgut, abdomen (at 4, 7, 10, 14 dpibm) and salivary glands (at 10, 14, 21 dpibm). The kinetics of ZIKV midgut infection intensity peak at 7 dpibm, with the Rock being significantly more permissive than the Orl strain at 4–10 dpibm (**Figures 1A–C**). The kinetics of disseminated infection, as measured by virus titers in the abdomen, were also similar for the two viruses, showing a peak in infection intensity at 14 dpibm (**Figures 1D–F**); the Rock strain was again more permissive to ZIKV infection at 10 and 14 dpibm. Salivary gland infection intensity and prevalence exhibited a gradual increase from 10 to 21 dpibm and showed a statistically non-significant trend toward being higher in the Rock strain (**Figures 1G–I**). Our study suggests that the kinetics of ZIKV infection intensity and prevalence differ between the *A. aegypti* Orl and Rock strains. While the Rock strain appeared to be more permissive to ZIKV infection at the stage of midgut and disseminated infection, virus infection intensity and prevalence of ZIKV in the salivary glands were similar between the two mosquito strains.

### ZIKV and DENV Infection-Responsive Transcriptomes Show Both Shared and Virus-Specific Responses

**FIGURE 2:**
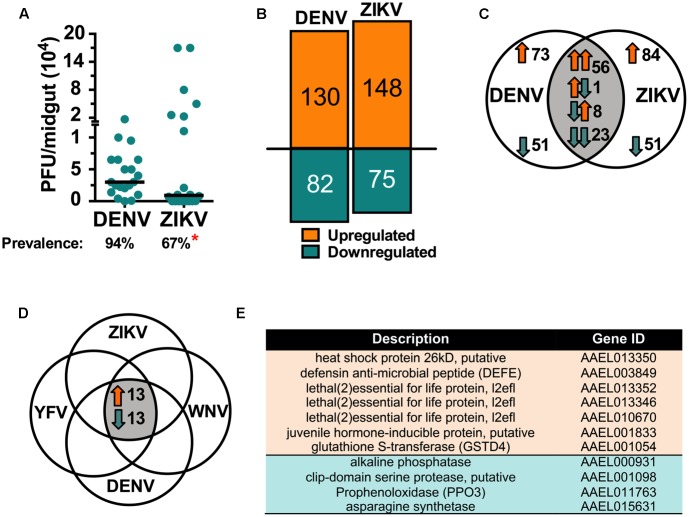
Comparative transcriptomic analysis of *A. aegypti* responses to ZIKV and DENV infection. RNA-seq analysis was performed on ZIKV- and DENV-infected Rock *A. aegypti* midguts at 7 dpibm. **(A)** PFU per midgut; bars represent the median for three independent experiments (*N* = 20). The prevalence (percentage) is shown below the graph, ^∗^*P* < 0.05; chi square test. **(B)** Total up- and downregulated genes. **(C)** Venn diagram showing the shared and uniquely regulated genes in ZIKV vs. DENV infected mosquitoes. **(D)** Venn diagram showing genes regulated in the same direction upon infection with YFV, WNV, DENV, or ZIKV (Colpitts et al., 2011). **(E)** List of the identified mosquito genes with known function (11 of 26): upregulated genes shown in orange, downregulated in blue. Upregulated genes with unknown function (AAEL004157, AAEL006305, AAEL000586, AAEL005986, AAEL009058, AAEL011110) and downregulated genes with unknown function (AAEL012644, AAEL009181, AAEL002046, AAEL007703, AAEL013812, AAEL002889, AAEL004022, AAEL008106, AAEL006834).

An organism’s transcriptome provides a detailed snapshot of its physiological state under a given condition. Therefore, transcriptomic infection-responses can be highly informative for understanding how virus infection affects the mosquito, and how the mosquito responds to counteract infection. While *A. aegypti* transcriptomic responses to DENV infection have been addressed in previous studies, using microarray-based technology, to the mosquito’s molecular responses to ZIKV infection were unknown. Here we used a more powerful RNAseq approach to study the mosquitoes’ responses, comparatively, to both DENV and ZIKV infection of the midgut tissue at 7 dpibm. This tissue and infection stage was chosen because it has proven to be highly informative with regard to characterizing immunity and other physiological responses to virus infection in previous studies with DENV. We used the more susceptible Rock strain to ensure high infection intensity and prevalence (**Figure 2**). Mosquito cohorts infected with each of the two viruses showed similar viral titers, but a lower prevalence of ZIKV-infected mosquitoes (67%, *P* = 0.0422) compared to those infected with DENV (**Figure 2A**). Infection by either ZIKV or DENV resulted in broad physiological responses entailing both common and unique expression signatures, reflecting possible differences in the interactions between the mosquito and the two viruses.

Infection with either virus resulted in a more prominent enrichment than depletion of transcripts; DENV and ZIKV infection led to an upregulation of 130 and 148 genes, respectively, and downregulation of 82 and 75 genes, respectively (**Figure 2B** and Supplementary Table S1). A comparison of the ZIKV and DENV infection-responsive transcriptomes showed 61% of the genes (135 of 223) to be uniquely regulated by ZIKV infection, whereas 35% (79 of 223) were regulated by both viruses in the same direction, and 4% in opposite direction (**Figure 2C**). A gene ontology functional analysis was then performed on the infection-responsive transcriptomes to determine the representation of specific functional groups and gene families. The defense response (biological processes category) (*P* < 0.01) and two redox activities (monooxygenase and oxidoreductase) in the molecular function category were significantly (*P* < 0.05) enriched in the regulated transcriptome repertoire (**Supplementary Figure S1**). The molecular function category also included numerous cytochrome P450 genes, which play roles in stress response and detoxification.

We then compared both our ZIKV and DENV infection-responsive transcriptomes with the published *A. aegypti* Rock strain YFV, WNV, and DENV2 infection-responsive transcriptomes (Colpitts et al., 2011). This analysis revealed 26 genes that were regulated in the same direction upon infection with all viruses; 13 genes were upregulated, and 13 genes were downregulated (**Figure 2D**). Only 11 of these genes have a predicted function and include factors associated with stress responses, immune responses, and enzymatic activity (**Figure 2E**).

### ZIKV Induces Mosquito Immune Responses, with a Bias toward the Toll Pathway

**FIGURE 3:**
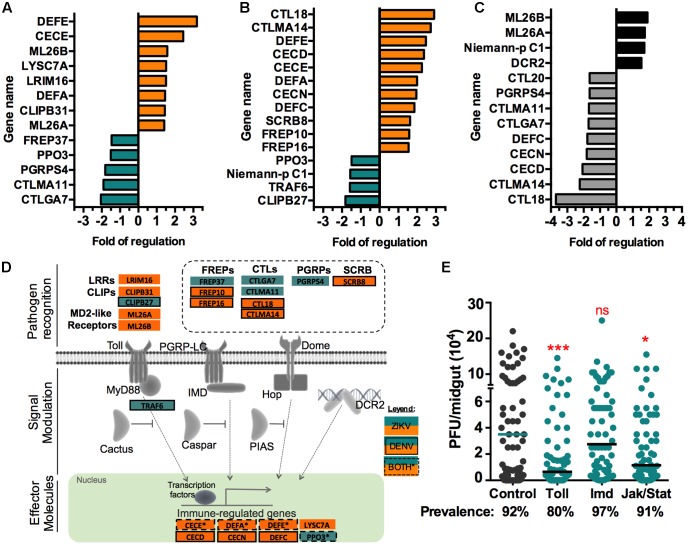
Infection-responsive immune genes. The fold change of putative immune gene transcript abundance upon midgut infection with **(A)** ZIKV or **(B)** DENV, as compared to non-infected blood-fed controls. Orange indicates upregulation, blue downregulation. **(C)** The fold difference in transcript abundance of immune genes between ZIKV- vs. DENV-infected midguts; black and gray indicates genes showing a higher transcript abundance in ZIKV and DENV infected midguts, respectively. **(D)** Schematic representation of the four main immune pathways (Toll, Imd, Jak/Stat, and RNAi). Boxes without lines represent ZIKV infection-responsive genes, boxes with solid lines represent DENV infection-responsive genes, and boxes with dashed lines represent genes regulated upon infection with either virus. **(E)** Immune pathway activation demonstrated through dsRNA-mediated silencing of the negative regulators of the Toll pathway (dsCactus, *N* = 65), Imd pathway (dsCaspar, *N* = 66), and Jak/Stat pathway (dsPIAS, *N* = 65). GFP dsRNA was used as a control (*N* = 62). ZIKV infection was measured as PFU in individual midguts at 7 days post-infection. The bars represent the median. ^∗^*P* < 0.05, ^∗∗^*P* < 0.01, ^∗∗∗^*P* < 0.001; Mann-Whitney test.

Data mining revealed a significant representation of the immune response ontology category in the ZIKV infection-responsive transcriptome. A closer assessment of immune genes, as classified in Waterhouse et al., 2007, identified 13 infection-responsive genes in ZIKV infected mosquitoes (**Figures 3A,D**). Some of these genes have predicted redundant immune functions, and other have been specifically associated with the mosquito immune pathways: Toll, immune deficiency (Imd), Jak/Stat, and RNAi. Six of the 13 (46%) infection-responsive immune-related genes are putatively linked to the Toll pathway, including Leucine rich repeat (LRRs)-containing proteins, clip-domain serine proteases (CLIPBs), myeloid differentiation 2-related lipid recognition protein (ML) receptors (MD2-like) and cecropin E (CECE) (Xi et al., 2008; Jupatanakul et al., 2014). The long peptidoglycan recognition proteins (PGRP-Ls) are frequently associated with the Imd pathway, but PGRPs have also been linked to the Toll pathway (Dziarski, 2004; Steiner, 2004). However, the specific role of each PGRP is not well understood, and different members may act as activators as well as negative regulators of the immune response (Waterhouse et al., 2007). The infection-responsive pathogen recognition receptor genes, including fibrinogen-related proteins (FREPs) and C-type lectins (CTLs) have not been specifically linked with any of the immune pathways. CECE is the only effector molecule most frequently associated with the Toll pathway; nevertheless, other CECs, defensins (DEF), and lysozymes (LYSC) can be regulated by both the Toll and Imd pathways (Zou et al., 2011). DENV-infected mosquitoes displayed a total of 15 regulated immune genes, with a greater representation of FREP and CTL family genes than in ZIKV-infected mosquitoes (**Figures 3B,D**). Interestingly, infection with either virus elicited upregulation of CECE, DEFA, and DEFE, all of which are common anti-microbial peptides. We also identified immune genes that were differentially regulated upon infection with ZIKV vs. DENV (**Figure 3C**). ZIKV infection specifically induced genes belonging to the ML family, which have been shown to influence DENV infection (Jupatanakul et al., 2014). ZIKV also induced expression of the Dicer-2 (dcr2) gene, a key factor of the antiviral RNAi immune pathway.

### Activation of the Toll and Jak/Stat Pathways Results in Suppression of ZIKV Infection

Transcriptome data can predict immune pathway activation but does not prove its implication in controlling virus infection. To assess the involvement of the innate immune pathways (Toll, Imd, and Jak/Stat) in regulating *A. aegypti’s* permissiveness to ZIKV infection, we used an RNA-mediated gene silencing approach to activate each of the pathways by depleting pathway-specific negative regulators of the Rock strain before feeding mosquitoes on ZIKV-infected blood. Activation of the Toll and Jak/Stat pathways, through depletion of Cactus and PIAS, respectively, resulted in a significantly lower infection intensity when compared to control mosquitoes treated with GPF dsRNA (*P* < 0.001 and *P* < 0.01, respectively) (**Figure 3E**). A modest decrease in prevalence was observed, but it was not statistically significant (*P* = 0.0539 and *P* = 0.8153). Activation of the Imd pathway through silencing of Caspar did not result in a significant modulation of ZIKV infection (*P* = 0.2334). These results show a similar pattern of immune pathway-specificity to that for DENV infection (Sim et al., 2013), where the Jak/Stat pathway was more potent in suppressing DENV, whereas we observed a greater potency of the Toll pathway in suppressing ZIKV.

### The vATPase and IMPDH Genes Act As Aedes Host Factors for Zika Infection

**FIGURE 4:**
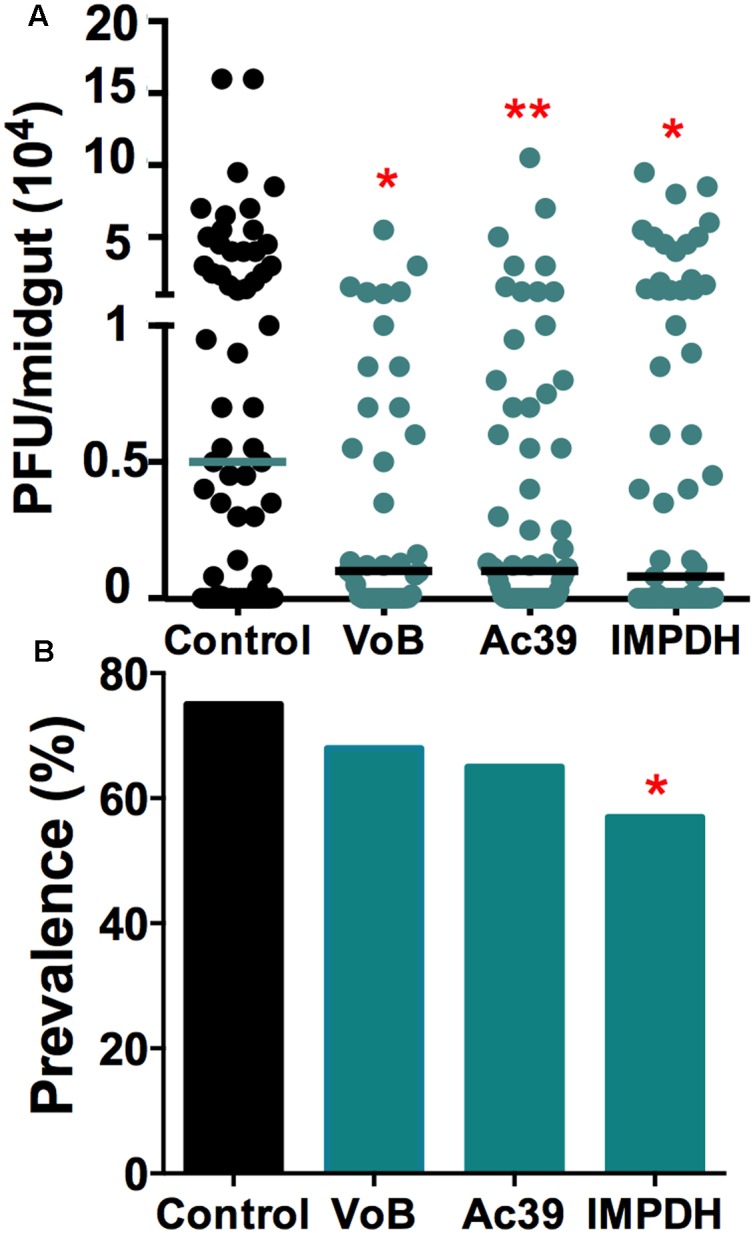
Mosquito host factors influence ZIKV infection. Genes encoding two subunits of the vATPase (VoB and Ac39) (*N* = 41, *N* = 65) and IMPDH (*N* = 63) were silenced upon ZIKV infection. **(A)** Each dot represents the PFUs per midgut in three independent experiments. The bars represent the median. ^∗^*P* < 0.05, ^∗∗^*P* < 0.01; Kruskal–Wallis test. **(B)** Prevalence (percentage) of infected mosquitoes. ^∗^*P* < 0.05; chi square test.

Arboviruses rely on numerous mosquito host factors for replication and infection, and we have previously confirmed that two subunits (VoB and Ac39) of the vacuolar ATPase (vATPase), and inosine-5’-monophosphate dehydrogenase (IMPDH), are essential DENV host factors (Kang et al., 2014). Others have confirmed the vATPase as a mammalian host factor for ZIKV (Savidis et al., 2016). The vATPase is required for the acidification of endosomes and viral genome release into the cytosol, and IMPDH is required for *de novo* RNA synthesis during viral replication. Silencing of these genes also resulted in reduced ZIKV infection intensity in the mosquito gut and a significant reduction in infection prevalence of IMPDH-depleted mosquitoes. These results show that that vATPase and IMPDH also serve as ZIKV host factors, and that the two viruses use some of these same mosquito factors to complete their replication cycle (**Figure 4**).

## Discussion

The recent ZIKV epidemic highlights the importance of studying the interactions of emerging arboviruses with their mosquito vectors. Despite their similarities in structure and transmission cycle, the outcomes of infection with ZIKV and DENV differ greatly in humans. Here, we performed a comparative study to address similarities and differences between the interactions of ZIKV and DENV with their common mosquito vector. The goal of this work was to initiate the elucidation of *A. aegypti* responses and immunity to ZIKV, while these features have been studied to some extent for DENV, to support the ongoing development of novel disease control strategies targeting the virus in the vector.

Compatibility between a virus and its mosquito vector is generally determined by virus host and restriction factors. These factors are broadly defined as mosquito proteins that support virus infection (have an agonistic function) or restrict virus infection (have an antagonistic function). Two *A. aegypti* strains, Rock and Orl, have previously been reported to differ in their vector competence for DENV (Sim et al., 2013). Here we show differences in permissiveness for ZIKV, at the midgut and disseminated stages of infection, with the Rock strain being a more permissive vector. Previous comparative transcriptome analyses between the Rock and Orl strains, in conjunction with RNAi-based studies of immune pathways, identified an infection bottleneck for DENV in the Orl strain. The study also showed a greater basal expression level of immune genes in the Orl strain, and implicated the Toll and Jak/Stat pathways in its lesser permissiveness to DENV infection (Sim et al., 2013).

DENV EIP is dependent on the mosquito strain, virus genotype, and environmental factors such as humidity and temperature. A peak of DENV2 infection intensity in the mosquito occurs at 7 dpibm (Salazar et al., 2007), which agrees with the tropism of ZIKV infection in our study. Several studies have described the *Aedes* transcriptome at different time-points during DENV infection. Others have indicated a modest immune gene regulation during the early stages of infection (1–4 dpibm), which is likely due to viral immune evasion and modulation mechanisms (Xi et al., 2008; Sim and Dimopoulos, 2010; Bonizzoni et al., 2012). Transcriptomic studies focusing on later infection time points (7–10 dpibm) documented the activation of the Toll and Jak/Stat pathways by DENV infection (Xi et al., 2008; Souza-Neto et al., 2009).

In the midgut tissue, that first encounters the pathogen, we found differences between the transcriptomic responses to ZIKV and DENV infection in the susceptible Rock strain at the 7 dpibm stage of peak infection. This indicates differences in the molecular interactions between the vector and each of the two viruses. We focused most of our data mining efforts on genes putatively being involved in the mosquito’s innate immune system. This immune system plays a crucial role in anti-viral defenses and therefore constitutes an infection bottleneck in some mosquito-virus combinations (Sanders et al., 2005; Franz et al., 2006; Galiana-Arnoux et al., 2006; Deddouche et al., 2008; Fragkoudis et al., 2008; Xi et al., 2008; Adelman et al., 2013; Sim et al., 2013). Genes belonging to different immune gene families were identified as ZIKV infection-responsive. The Clip-domain serine protease gene, CLIPB31, was regulated upon ZIKV infection. The CLIP gene family comprises over 60 members, some of which have been associated with immune pathway activation as well as melanization and lytic effector mechanisms (Xi et al., 2008; Kanost and Jiang, 2015). Interestingly, at least four CLIP genes (CLIPB13B, CLIPB46, CLIPB5, CLIPC2) have been shown to be induced upon Toll pathway activation (Xi et al., 2008). The transcript abundance of CLIPB31 is also significantly increased in mosquitoes infected with the DENV-blocking endosymbiont *Wolbachia* (Rancès et al., 2012), pointing at a possible link between immune system modulation by the bacterium and virus suppression.

Antimicrobial peptide (AMPs) genes encode short peptides that interact with the pathogen to mediate their elimination through mechanisms that include lysis and membrane disruption (Kaushal et al., 2016; Zheng et al., 2017). Here we found that AMPs such as LYSCs, DEFs, and CECs, were induced upon ZIKV infection, but their potential role in antiviral defense has not yet been studied (Xi et al., 2008; Zou et al., 2011). We identified members of the ML receptors in the ZIKV infection-responsive transcriptome. The mammalian MD-2 mediates Toll pathway activation through the TLR4 receptor (Stokes et al., 2015). The role of MD-2–like proteins in mosquito immunity is not well understood, but the *Anopheles gambiae* ML1 gene has been linked to both antibacterial and anti-*Plasmodium* defenses, and a *Drosophila* ML gene has also been linked to Imd pathway activation (Dong et al., 2006; Shi et al., 2012; Sandiford et al., 2015). The *A. aegypti* genome contains 26 ML-like gene of which ML26A and ML26B were upregulated upon ZIKV infection. These two genes have been shown to be upregulated in DENV-resistant *A. aegypti* at 18 dpibm (Behura et al., 2011). Another study has shown that ML13 and ML33 are induced by DENV infection in susceptible mosquitoes, and ML33 acts as an agonist for DENV (Jupatanakul et al., 2014). The fibrinogen domain -containing gene FREP37 was regulated upon ZIKV infection, and this gene has also been shown to be downregulated upon infection with YFV and DENV2 (Behura et al., 2011; Colpitts et al., 2011). DENV-infected mosquitoes showed upregulation of the FREP10 and FREP16 genes that have been previously associated with anti-bacterial responses (Rancès et al., 2012; Ye et al., 2013). FREPs belong to a large gene family, and have been shown to function as putative pathogen recognition receptors for both bacteria and *Plasmodium* (Dong and Dimopoulos, 2009).

Our transcriptomic study revealed a bias toward the Toll pathway upon ZIKV infection, and we further confirmed a role for this pathway in suppressing ZIKV through RNAi-based reverse genetic assays. We also demonstrated that activation of the Jak/Stat pathway contributes to ZIKV suppression. Both the Toll and Jak/Stat pathways have previously been shown to suppress DENV infection in the midgut tissue, thereby corroborating a major role for these immune pathways in suppressing flaviviruses (Sanders et al., 2005; Xi et al., 2008; Fragkoudis et al., 2009; Souza-Neto et al., 2009; Ramirez and Dimopoulos, 2010; Tchankouo-Nguetcheu et al., 2010; Colpitts et al., 2011; Sim et al., 2013). A recent study using a transgenic approach showed that activation of the Jak/Stat pathway in the mosquito’s fatbody (contained in the abdomen and thorax) did not affect susceptibility to ZIKV infection while it strongly suppressed DENV infection. This discrepancy with our RNAi-based assays is interesting and suggests possible differences in the immune pathway’s spatial (tissue) and temporal antiviral specificity (Jupatanakul et al., 2017).

The RNAi pathway cannot be transiently activated through gene silencing since a negative regulator has yet not been identified, but partial inhibition of the pathway using RNAi to silence dicer-2 (dcr2) increases the susceptibility of refractory Orl strain mosquitoes to DENV infection (Sim et al., 2013). We found that dcr2 expression was significantly higher in ZIKV-infected than in DENV-infected mosquito midguts, suggesting that the RNAi pathway is likely to be involved in suppressing ZIKV infection.

The functional annotation of *A. aegypti* immune genes is based on phylogenomic analysis using the *Drosophila melanogaster* immune gene repertoire, as well as those of other disease-vector mosquitoes (Waterhouse et al., 2007). Many of our ZIKV and DENV infection-responsive genes were of unknown function and are therefore likely to represent additional factors of the mosquito’s antiviral defense system.

Of the 88 genes that responded to infection with both viruses (42% of the ZIKV infection-responsive transcriptome), 9 were regulated in opposite directions upon infection with DENV vs. ZIKV. Interestingly, five of these were trypsin genes that were downregulated in DENV- and upregulated in ZIKV-infected midguts. Trypsins have previously been associated with the modulation of DENV infection in *A. aegypti* midguts (Brackney et al., 2010). This suggests that both viruses have the potential to differentially modulate the mosquito’s digestive processes.

By comparative analysis of the ZIKV, DENV, YFV, and WNV infection-responsive transcriptomes, derived from our and a previous study, we identified 26 genes that were regulated in the same direction during infection of *A. aegypti* with the four viruses (Colpitts et al., 2011). Despite possible differences in experimental procedures between the studies, this finding suggests that at least some of these genes are part of the mosquito’s general response to virus infection. Three of them represent potential pan-flavivirus infection-responsive immune genes [DEFE, a putative CLIP, and prophenoloxidase 3 (PPO3)]. We also identified three lethal (2) essential for life (l(2)efl) genes, which are relatively unstudied but encode for small heat shock proteins (Kurzik-Dumke and Lohmann, 1995). Heat shock proteins have been linked with apoptosis, a process that is associated with the mosquito’s defense against virus infection, and previous studies have suggested their possible interaction with factors of the antiviral Toll pathway (Guo et al., 2010; Gonda et al., 2012). While current research on transmission-blocking approaches are mostly focused on targeting a single pathogen the identification and characterization of mosquito defense systems that can control multiple types of viruses is of particular importance for the development of pan-flavivirus transmission-blocking strategies (Shin et al., 2003; Bian et al., 2005; Adelman et al., 2008; Dong et al., 2011; Zou et al., 2011; Jupatanakul et al., 2017; Simões et al., 2017).

As part of our comparative study of the molecular interactions between the two viruses and the *A. aegypti* vector, we also tested the possible involvement of two DENV host factors in ZIKV infection (Kang et al., 2014). Silencing of two vATPase subunits (VoB and Ac39) and IMPDH revealed that they also play ZIKV host factor functions. Interestingly, a recent study has shown that the mammalian vATPase is a ZIKV host factor (Savidis et al., 2016), indicating conserved components of the cellular machinery involved in sustaining viral replication in both hosts. Virus host factors represent potent transmission-blocking targets through multiple approaches, including transgenic gene deletion, small molecule inhibitors and transmission-blocking vaccines (Bowman et al., 1988; Diamond et al., 2002; Kang et al., 2014; Liu et al., 2014; Dong et al., 2015; Sandiford et al., 2015; Londono-Renteria et al., 2016).

In summary, we report that ZIKV and DENV have a similar tropism and ability to establish infection in low and high infection-permissive *A. aegypti* strains. The study generated new insights regarding the molecular responses of the vector mosquito to infection with ZIKV and DENV, and shows that ZIKV infection elicits quite a different transcriptome response, although many immune genes are similarly regulated by infection with both viruses. The major innate immune pathways, Toll and Jak/Stat, play roles in suppressing both viruses, and vATPase and IMPDH likely represent general flavivirus host factors in both mosquitoes and humans. This study also identifies numerous mosquito genes that are likely to participate in responding to, and controlling, virus infection. Our study has significantly contributed toward the gradually growing knowledge of mosquito–virus interactions, aiding in the further focus on specific factors, defense systems and host factors that can be used to develop novel flavivirus transmission blocking strategies. For example, the spread pathogen-resistance genes in mosquito population has in recent years gained further interest through the development of gene-drive systems (Franz et al., 2006, 2014; Windbichler et al., 2011; Gantz et al., 2015; Jupatanakul et al., 2017). The development of virus resistant mosquitoes will require knowledge on virus restriction factors and infection-responsive promoters to express the antiviral genes. Furthermore, multiple research efforts are addressing the inhibition of mosquito-encoded virus agonists (host factors) through small molecule inhibitors, transmission-blocking vaccines and gene deletion (Bowman et al., 1988; Diamond et al., 2002; Kang et al., 2014; Liu et al., 2014; Dong et al., 2015; Londono-Renteria et al., 2016). The current lack of protective vaccines and drugs for most arboviruses, and the emergence of mosquito resistance to insecticides, have created an increasing need for additional complementary disease control strategies.

## Materials and Methods

### Ethics Statement

This study was carried out in accordance with the recommendations in the Guide for the Care and Use of Laboratory Animals of the National Institutes of Health, the Animal Care and Use Committee of the Johns Hopkins University and the institutional Ethics Committee (permit number: M006H300). Mice were only used for mosquito rearing. Commercial, anonymous human blood was used for virus infection assays in mosquitoes, and informed consent was therefore not applicable.

### Cell Culture and Mosquito Rearing

The *A. albopictus* cell line (C6/36) was maintained in MEM (Gibco) supplemented with 10% FBS, 1% L-glutamine, 1% NEA, and 1% penicillin/streptomycin. Baby hamster kidney cells (BHK-21), clone 15, were maintained in DMEM (Gibco) supplemented with 10% FBS, 1% L-glututamine, 1% penicillin/streptomycin, and 5 μg/mL plasmocin (Invivogen). C6/36 cells and BHK-21 cells were incubated with 5% CO_2_ at 32°C and 37°C, respectively. *A. aegypti* mosquitoes were maintained on 10% sucrose solution at 27°C and 80% relative humidity with a 14:10 h light:dark cycle.

### ZIKV or DENV Infections and Virus Titration

ZIKV strain FSS13025 or DENV NGC were propagated in C6/36 cells, and titers were determined by plaque assay on Vero cells for ZIKV or BHK-21 for DENV. ZIKV or DENV were propagated in C6/36 cells for 6 days; virus was then harvested and mixed with a sterile 1% solution at pH 7.1 (2.18 M sucrose, 38 mM KH_2_PO4, 72 mM K_2_HPO_4_, 60 mM L-glutamic acid), and stored at -80°C for future experiments. The virus suspension was mixed 1:1 with commercial human blood and supplemented with 10% human serum and 1% 10 mM ATP. Mosquitoes were infected via an artificial membrane feeder at 37°C for 30 min. Midguts, abdomens (without midguts), and salivary glands were dissected, individually collected, and stored at -80°C until used for plaque assays. Virus titration was performed as described in (Sim et al., 2013), plates were incubated for 4–5 days, fixed and stained with methanol/acetone and 1% crystal violet mixture, washed, and the plaque-forming units counted.

### RNA Sequencing and Data Analysis

#### Sample Preparation

*Aedes aegypti* Rockefeller strain mosquitoes were fed with a naïve blood meal containing non-infected cell culture or infected with 1 × 10^7^ plaque-forming units (PFU)/mL of DENV2 NGC or ZIKV FSS13025 for 7 days. The midguts were dissected, pools of 20 midguts were collected per group, and three independent biological replicates were assayed. RNA was extracted with the Quick-RNA MiniPrep kit^TM^ (Zymo Research). An Illumina sequencing library was constructed for each of the nine biological samples according to the manufacturer and sequenced on the Illumina NextSeq 500 sequencer. Compressed FASTQ files were extracted, mapped, and aligned to the mosquito transcriptome into the QIAGEN CLC Genomics Server platform^1^. The annotated *A. aegypti* (NCBI Taxonomy ID: 1424507) reference transcriptome from the ENSEMBL metazoan database was used for annotation. Across the sequenced samples, between 82 and 85% of the reads aligned to the 17,478 annotated genes were used to assign FPKM (Fragments Per Kilobase of transcript per Million mapped reads) values to those genes. These nine FPKM datasets were then imported into the Partek Genomics Suite v6.6 analytic platform (Partek Inc. St Louis, MO, United States) for further processing and comparison of their experimental classes: ZIKV infection, DENV infection, and naïve blood controls. These raw datasets were quantile normalized, values were converted to log_2_ notation, and two-tailed one-way ANOVAs was conducted comparing infected mosquito samples to controls and to themselves. These comparisons yielded each gene’s relative expression, as a fold change, and that change’s statistical significance, as its *p*-value. These ANOVA results were then imported for further analysis and graphic display into the Spotfire Genomics Suite v9.1.2 platform (TIBCO Spotfire, Boston, MA, United States). The comparisons were filtered to exclude transcripts whose minimum (infected or Control) normalized Log_2_(FPKM) value was less than -0.6 to avoid unreliable fold changed caused by stochastic noise in too low signal values. Since the remaining approximately 6K log2 fold changes showed normal distributions, their standard deviation changes from the mean of no change were established and used to set a 2 SD thresholds for significant differential expression (linear fold changes of 1.40, 1.49, and 1.54 absolute value for ZIKV vs. Cont, DENV vs. Cont, and ZIKV vs. DENV respectively). RNAseq data are available from NCBI GEO with accession number GSE96605.

### Pan-Flavivirus Transcriptome Analysis

We used the available transcriptome data of (Colpitts et al., 2011) for DENV, YFV, and WNV, significantly regulated genes with *P* < 0.05, log_2_ fold change > 1 or < -1, regulated in the same direction for at least one-time point per virus. Selected genes were then compared to our RNAseq transcriptome selecting only genes that were significantly regulated in the same direction for both, ZIKV and DENV.

### dsRNA-Mediated Gene Silencing

The *cactus, caspar, and PIAS* genes ware depleted from adult female mosquitoes using established RNAi methodology (Sim et al., 2013). Assays were repeated three times, using *GFP* dsRNA as a control. Gene silencing was verified at 3 days post-injection of RNA extracted from five whole mosquitoes per biological replicate, and two technical replicates were analyzed by qRT-PCR (**Supplementary Figure S2**). The ribosomal protein S7 gene was used to standardize and verify gene silencing. The primers used to produce PCR amplicons for dsRNA synthesis and qRT-PCR are given in Supplementary Table S2.

### Statistical Analysis

The Graphpad Prism 6 (Graphpad Prism^®^) software package was used to perform statistical analyses. The particular test used is indicated in the legend of each respective figure. See Supplementary Table S3 for a summary of the statistics.

## Author Contributions

YA-R, NJ, and GD conceived experiments; YA-R, HM, SK, JC, and NJ performed experiments. YA-R and GD analyzed the data obtained and wrote the manuscript.

## Conflict of Interest Statement

The authors declare that the research was conducted in the absence of any commercial or financial relationships that could be construed as a potential conflict of interest.

## Footnotes

1 https://www.qiagenbioinformatics.com

## Supplementary Material

**FIGURE S1 |** Gene ontology functional analysis. Biological process, cellular component, molecular function, and immune system terms were analyzed for all significantly regulated genes using ClueGO, a Cytoscape plugin, and the *A. aegypti* genome was used as background. **(A)** Bars show the percent of genes per term, and those with similar functional categories have the same color. **(B)** Pie chart graph shows total genes represented per term, the identified term was based on highest enrichment. Asterisk represents significantly enriched terms. ^∗^*P* < 0.05, ^∗∗^ *P* < 0.01, Bonferroni test. Only gene terms with more than three genes per category are presented in the graph. **(C)** List of genes per term.

**FIGURE S2 |** Silencing efficiency. qRT-PCR of silenced genes *cactus* (Toll), *caspar* (Imd), *and PIAS* (Jak/Stat). **(A)** Immune genes. **(B)** Host factors. Bars represent SEM of three independent experiments.
