# Supplementary figures and images for: Aedes aegypti Molecular Responses to Zika Virus: Modulation of Infection by the Toll and Jak/Stat Immune Pathways and Virus Host Factors
Source: Front Microbiol. 2017 Oct 23;8:2050. doi: 10.3389/fmicb.2017.02050 (PMC5660061; doi:10.3389/fmicb.2017.02050)

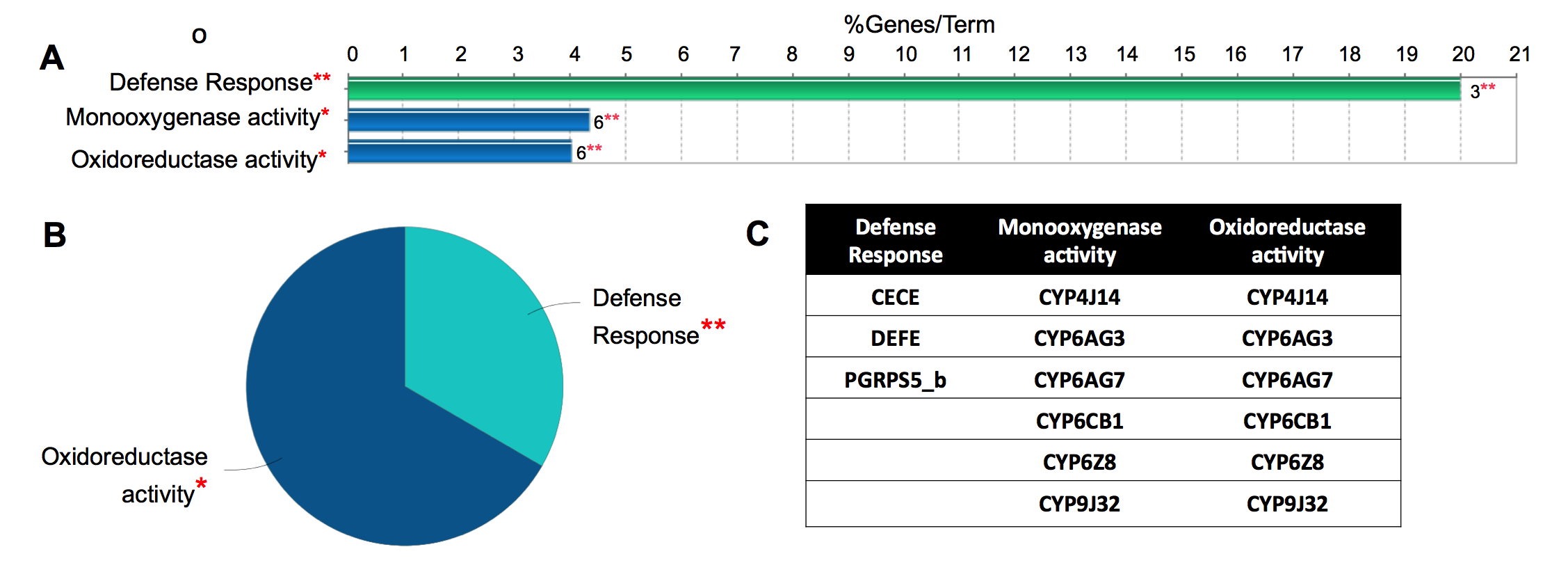

Supplement: Supplementary file 1 [file Image_1.TIFF]

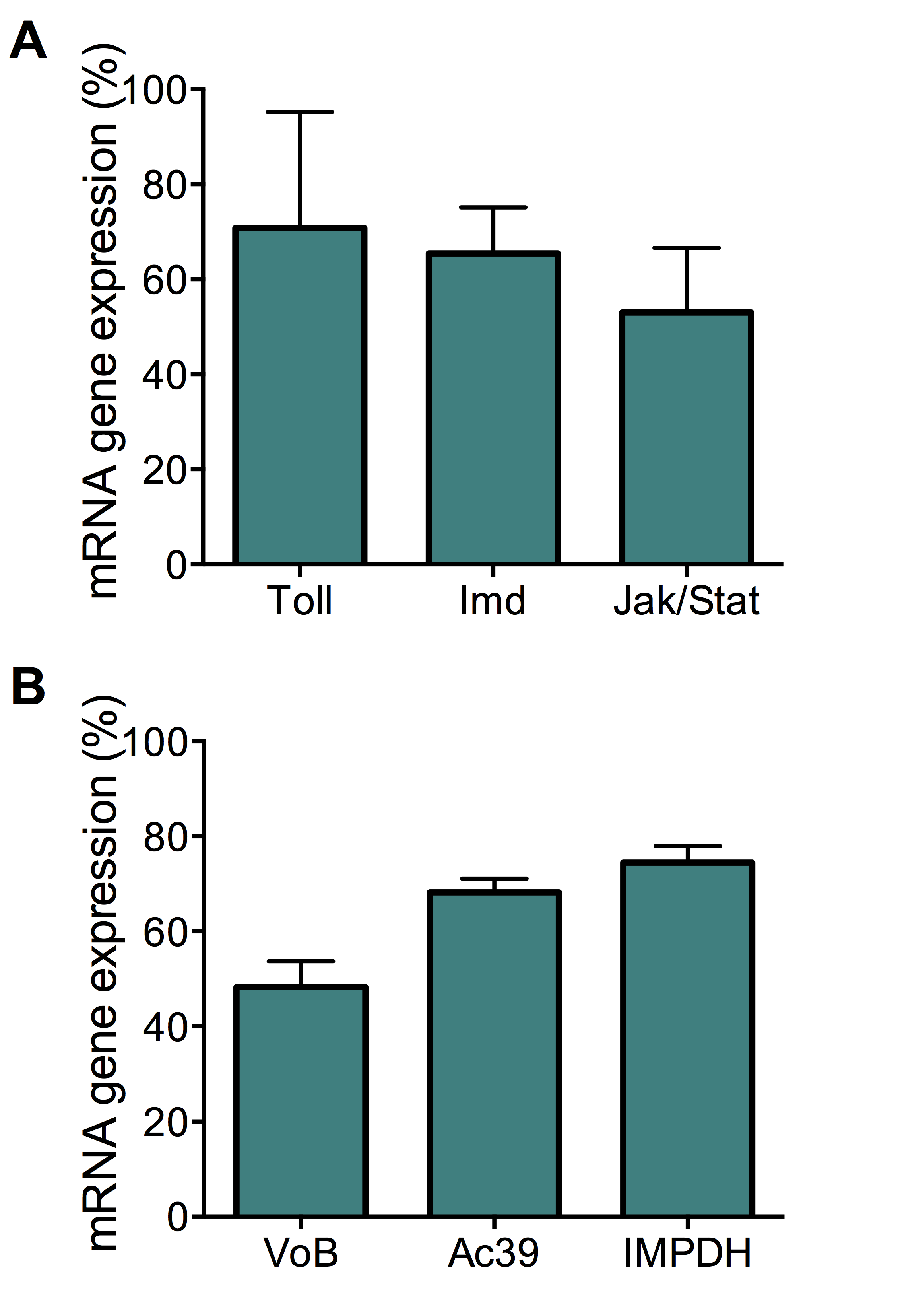

Supplement: Supplementary file 2 [file Image_2.TIFF]
