# Supplementary material for: Aedes aegypti Molecular Responses to Zika Virus: Modulation of Infection by the Toll and Jak/Stat Immune Pathways and Virus Host Factors
Source: Front Microbiol. 2017 Oct 23;8:2050. doi: 10.3389/fmicb.2017.02050 (PMC5660061; doi:10.3389/fmicb.2017.02050)
Supplement: Supplementary file 3 [file Table_1.PDF]

Table S1: *Ae. aegypti* midgut transcriptome 7 days post-infection. Values represent the fold change of DENV- or ZIKV-regulated genes compared to non-infected control.

Table 1A: DENV unique downregulated genes

Column ID	Description	DV FC
**AAEL006498**	long wavelength sensitive opsin (GPROP1)	-4.57
**AAEL013284**	serine-type endopeptidase, putative	-3.03
**AAEL013714**	trypsin, putative	-2.89
**AAEL006883**	conserved hypothetical protein	-2.44
**AAEL008336**	snail protein, putative	-2.15
**AAEL002467**	conserved hypothetical protein	-2.12
**AAEL006546**	hypothetical protein	-1.99
**AAEL017046**	Matrix metalloproteinase, putative	-1.97
**AAEL017549**	GPCR Orphan/Putative Class A Family (GPRNNA14)	-1.86
**AAEL014542**	hypothetical protein	-1.86
**AAEL000019**	conserved hypothetical protein	-1.82
**AAEL017597**	28S rRNA	-1.82
**AAEL007993**	Clip-Domain Serine Protease family B (CLIPB27)	-1.81
**AAEL002495**	conserved hypothetical protein (mucin-like protein)	-1.80
**AAEL000859**	hypothetical protein	-1.79
**AAEL006256**	ATPase subunit, putative	-1.74
**AAEL003448**	GPCR Orphan/Putative Class A Family (GPRNNA13)	-1.71
**AAEL008776**	conserved hypothetical protein	-1.70
**AAEL001667**	multicopper oxidase	-1.68
**AAEL007191**	amino acid transporter	-1.67
**AAEL008913**	amino acid transporter	-1.65
**AAEL013717**	sphingomyelin phosphodiesterase	-1.65
**AAEL017334**	conserved hypothetical protein (chitin-binding domain type 2)	-1.64
**AAEL006811**	cytochrome P450 (CYP9J8)	-1.63
**AAEL004986**	smg-7 (suppressor with morphological effect on genitalia protein 7)	-1.63
**AAEL012921**	ribosomal protein L36, putative	-1.59
**AAEL013944**	hypothetical protein	-1.59
**AAEL001906**	pnuts protein	-1.59
**AAEL001992**	hypothetical protein	-1.59
**AAEL011363**	TOLL pathway signalling TNF Receptor-Associated Factor (TRAF6)	-1.58
**AAEL010540**	alpha-amylase	-1.58
**AAEL007555**	acyl-coa dehydrogenase	-1.57
**AAEL013909**	hypothetical protein	-1.57
**AAEL009531**	niemann-pick C1	-1.56
**AAEL015315**	malate synthase	-1.56
**AAEL002588**	conserved hypothetical protein	-1.54
**AAEL006198**	hypothetical protein	-1.53
**AAEL017034**	Hypothetical protein	-1.53
**AAEL001359**	hypothetical protein	-1.52
**AAEL009219**	predicted protein	-1.52
**AAEL004397**	conserved hypothetical protein	-1.52
**AAEL014622**	conserved hypothetical protein	-1.52
**AAEL002733**	neuropeptide F Precursor (NPF)	-1.51
**AAEL014913**	pyruvate kinase	-1.51
**AAEL012757**	kynurenine 3-monooxygenase	-1.49

**AAEL012776**	protease m1 zinc metalloprotease	-1.49
**AAEL010912**	dipeptidyl-peptidase	-1.49
**AAEL008163**	protease m1 zinc metalloprotease	-1.49
**AAEL007892**	xaa-pro aminopeptidase	-1.49
**AAEL003313**	alkaline phosphatase	-1.49
**AAEL008470**	hypothetical protein	-1.48

Table 1B: DENV unique upregulated genes

Column ID	Description	DENV FC
**AAEL005482**	C-Type Lectin (CTL18)	2.90
**AAEL014382**	C-Type Lectin-mannose binding (CTLMA14)	2.72
**AAEL010667**	lethal(2)essential for life protein, l2efl	2.47
**AAEL018349**	Cecropin D (CECD)	2.36
**AAEL004031**	conserved hypothetical protein	2.25
**AAEL008409**	p15-2a protein, putative	2.10
**AAEL000621**	Cecropin N (CECN)	1.96
**AAEL003029**	hypothetical protein	1.94
**AAEL005315**	conserved hypothetical protein	1.92
**AAEL003832**	Defensin C (DEFC)	1.86
**AAEL003869**	conserved hypothetical protein	1.84
**AAEL010075**	oxidoreductase	1.82
**AAEL011371**	conserved hypothetical protein	1.82
**AAEL014891**	cytochrome P450 (CYP6P12)	1.81
**AAEL003641**	sodium/shloride dependent amino acid transporter	1.74
**AAEL000401**	conserved hypothetical protein	1.69
**AAEL006605**	Juvenile hormone-inducible protein, putative	1.69
**AAEL003270**	conserved hypothetical protein	1.69
**AAEL013711**	conserved hypothetical protein	1.68
**AAEL000419**	conserved hypothetical protein	1.68
**AAEL013658**	conserved hypothetical protein	1.67
**AAEL003523**	hypothetical protein	1.66
**AAEL005001**	aquaporin	1.65
**AAEL004369**	alpha-glucosidase	1.65
**AAEL003405**	conserved hypothetical protein	1.65
**AAEL005926**	ER lumen protein retaining receptor	1.64
**AAEL004665**	conserved hypothetical protein	1.63
**AAEL009945**	conserved hypothetical protein	1.63
**AAEL009611**	conserved hypothetical protein	1.63
**AAEL008259**	conserved hypothetical protein	1.63
**AAEL000227**	Class B Scavenger Receptor, CD36 domain (SCRB8)	1.62
**AAEL011278**	apolipoprotein D, putative	1.62
**AAEL013491**	short-chain dehydrogenase	1.61
**AAEL005040**	conserved hypothetical protein	1.60
**AAEL001757**	iduronate 2-sulfatase precursor	1.60
**AAEL004043**	conserved hypothetical protein	1.59
**AAEL005819**	l-allo-threonine aldolase	1.59
**AAEL013591**	dynactin, p27-subunit, putative	1.58
**AAEL008646**	fibrinogen and fibronectin	1.58
**AAEL001366**	conserved hypothetical protein	1.58
**AAEL009448**	conserved hypothetical protein	1.58
**AAEL015454**	conserved hypothetical protein	1.57
**AAEL017457**	Catalytic activity, serine-type endopeptidase activity, protelysis	1.57
**AAEL008651**	conserved hypothetical protein	1.57
**AAEL004254**	hypothetical protein	1.57
**AAEL004809**	conserved hypothetical protein	1.56
**AAEL002385**	Carboxy/choline esterase Alpha Esterase (CCEAE3B)	1.55
**AAEL015104**	Trypsin, putative	1.55
**AAEL011088**	conserved hypothetical protein	1.55

**AAEL008802**	conserved hypothetical protein	1.55
**AAEL013560**	mitochondrial ribosomal protein, L50, putative	1.55
**AAEL003967**	calpain, putative	1.55
**AAEL009060**	hypothetical protein	1.55
**AAEL009922**	conserved hypothetical protein	1.55
**AAEL011633**	fibrinogen and fibronectin	1.54
**AAEL009270**	hypothetical protein	1.54
**AAEL018415**	Sphinx 2	1.54
**AAEL006318**	short-chain dehydrogenase	1.54
**AAEL006203**	Juvenile hormone-inducible protein, putative	1.54
**AAEL010468**	DNA-directed RNA polymerase I, 12kD-subunit, putative	1.53
**AAEL005232**	cyclin-dependent kinases regulatory subunit. putative	1.53
**AAEL015526**	hypothetical protein	1.53
**AAEL003113**	conserved hypothetical protein	1.52
**AAEL010072**	hypothetical protein	1.52
**AAEL004425**	ctg4a	1.52
**AAEL004188**	conserved hypothetical protein	1.52
**AAEL003264**	conserved hypothetical protein	1.51
**AAEL017228**	hypothetical protein	1.51
**AAEL006365**	trypsin-alpha, putative	1.51
**AAEL001775**	conserved hypothetical protein	1.50
**AAEL006030**	hypothetical protein	1.50
**AAEL004042**	conserved hypothetical protein	1.50
**AAEL004488**	conserved hypothetical protein	1.50

Table 1C: Zika unique downregulated genes

Column ID	Description	ZIKV FC
**AAEL009936**	conserved hypothetical protein	-2.71
**AAEL000200**	conserved hypothetical protein	-2.24
**AAEL012184**	glutaminyl-peptide cyclotransferase	-2.18
**AAEL013338**	lethal(2)essential for life protein, l2efl	-2.08
**AAEL017265**	C-Type Lectin- galactose binding (CTLGA7)	-2.05
**AAEL014794**	mitochondrial transcription factor A, putative	-1.99
**AAEL000543**	C-Type Lectin-mannose binding (CTLMA11)	-1.91
**AAEL012657**	syntaxin, putative	-1.86
**AAEL007557**	asparagine synthetase	-1.85
**AAEL017491**	hypothetical protein	-1.84
**AAEL017977**	heat shock protein HSP70 (HSP70Ca)	-1.84
**AAEL017056**	Peptidoglycan Recognition Protein Short (PGRPS4)	-1.81
**AAEL014515**	metalloproteinase, putative	-1.80
**AAEL003816**	hypothetical protein	-1.78
**AAEL009748**	2-hydroxyphytanoyl-coa lyase	-1.64
**AAEL017904**	5.8S rRNA	-1.63
**AAEL001724**	GPCR Orphan/Putative Class B Family (GPRNNB3)	-1.63
**AAEL003803**	conserved hypothetical protein	-1.63
**AAEL005889**	hypothetical protein	-1.62
**AAEL010169**	conserved hypothetical protein	-1.61
**AAEL011190**	conserved hypothetical protein	-1.61
**AAEL002307**	Leucine-rich transmembrane protein	-1.60
**AAEL017234**	Oxygen transporter activity, transport.	-1.59
**AAEL005331**	hypothetical protein	-1.57
**AAEL002347**	serine-type enodpeptidase, putative	-1.56
**AAEL009206**	organic cation transporter	-1.56
**AAEL015493**	conserved hypothetical protein	-1.55
**AAEL014188**	serine-type enodpeptidase, putative	-1.54
**AAEL017974**	heat shock protein HSP70 (HSP70Ab)	-1.54
**AAEL008304**	hypothetical protein	-1.52
**AAEL012316**	arsenical pump-driving ATPase (ASNA1-2)	-1.52
**AAEL009021**	peptidylprolyl isomerase	-1.49
**AAEL012592**	conserved hypothetical protein	-1.49
**AAEL008931**	inward-rectifying potassium channel (Kir2B)	-1.49
**AAEL014850**	conserved hypothetical protein	-1.48
**AAEL000080**	phosphoenolpyruvate carboxykinase	-1.47
**AAEL010257**	conserved hypothetical protein	-1.47
**AAEL011007**	fibrinogen and fibronectin	-1.46
**AAEL006902**	serine-type enodpeptidase, putative	-1.46
**AAEL002976**	Aspartyl beta-hydroxylase, putative	-1.44
**AAEL013554**	cytochrome P450 (CYP4J14)	-1.44
**AAEL014654**	hypothetical protein	-1.43
**AAEL011057**	DNApair protein complementing XP-A cells, putative	-1.43
**AAEL013257**	conserved hypothetical protein	-1.43
**AAEL003345**	argininosuccinate lyase	-1.42
**AAEL001274**	hypothetical protein	-1.42
**AAEL018543**	28S rRNA	-1.42
**AAEL011175**	alkaline phosphatase	-1.42
**AAEL014292**	40S ribosomal protein S24	-1.41

**AAEL001766**	Leucine-rich transmembrane protein, putative	-1.41
**AAEL014498**	hypothetical protein	-1.41

Table 1D: Zika unique upregulated genes

Column ID	Description	ZIKV FC
**AAEL002875**	conserved hypothetical protein	17.63
**AAEL013127**	conserved hypothetical protein	3.38
**AAEL013713**	trypsin	2.90
**AAEL013577**	conserved hypothetical protein	2.87
**AAEL007601**	trypsin	2.46
**AAEL012189**	multidrug resistance protein 1 (atp-binding cassette C1)	2.44
**AAEL012395**	ATP-binding cassette transporter	2.35
**AAEL013103**	conserved hypothetical protein	2.24
**AAEL013021**	hypothetical protein	2.05
**AAEL017973**	heat shock protein HSP70 (HSP70Aa)	2.00
**AAEL008308**	conserved hypothetical protein	1.84
**AAEL010776**	carboxypeptidase	1.82
**AAEL006953**	conserved hypothetical protein	1.78
**AAEL015445**	cysteine dioxygenase	1.74
**AAEL010163**	conserved hypothetical protein	1.71
**AAEL001701**	serine-type enodpeptidase, putative	1.71
**AAEL006562**	acid phosphatase	1.71
**AAEL015114**	conserved hypothetical protein	1.71
**AAEL010620**	conserved hypothetical protein	1.71
**AAEL018688**	tRNA-Leu	1.70
**AAEL018514**	Arthropod 7SK RNA	1.68
**AAEL010782**	carboxypeptidase	1.67
**AAEL018527**	Metazoan signal recognition particle RNA	1.67
**AAEL017172**	ribokinase , catalyses the phosphorlation of ribose to ribose-5phosphate using ATP.	1.65
**AAEL018658**	NADH dehydrogenase subunit 2 (ND2)	1.64
**AAEL009684**	hypothetical protein	1.64
**AAEL010920**	conserved hypothetical protein	1.62
**AAEL001863**	zinc carboxypeptidase	1.62
**AAEL007416**	cysteine dioxygenase	1.61
**AAEL002388**	zinc finger protein	1.61
**AAEL008279**	conserved hypothetical protein	1.59
**AAEL009131**	cytochrome P450 (CYP6Z8)	1.59
**AAEL013835**	conserved hypothetical protein	1.58
**AAEL009844**	conserved hypothetical protein	1.57
**AAEL018671**	NADH dehydrogenase subunit 3 (ND3)	1.56
**AAEL018627**	28S rRNA	1.56
**AAEL003603**	transcription factor, putative	1.55
**AAEL009130**	cytochrome P450 (CYP6Z7)	1.54
**AAEL005561**	plasma membrane calcium-transporting atpase 3 (pmca3)	1.54
**AAEL008385**	Histone H2A, putative	1.53
**AAEL013276**	acid phosphatase	1.52
**AAEL010100**	C-type lysozyme (LYSC7A)	1.52
**AAEL005723**	conserved hypothetical protein	1.51
**AAEL008658**	leucine-rich immune protein (TM) (LRIM16)	1.51
**AAEL017167**	GPCR HE6-like Family (GPRHE6)	1.50
**AAEL007951**	glutathione transferase (GSTE2)	1.50
**AAEL000727**	hypothetical protein	1.50
**AAEL004632**	conserved hypothetical protein	1.49

**AAEL015618**	conserved hypothetical protein	1.49
**AAEL013093**	valacyclovir hydrolase	1.49
**AAEL017241**	Hypothetical protein	1.48
**AAEL006242**	beta chain spectrin	1.48
**AAEL009104**	vacuolar protein sorting-associated protein (vps13)	1.48
**AAEL009237**	glycoside hydrolases	1.47
**AAEL006381**	sphingomyelin phosphodiesterase	1.47
**AAEL013090**	hypothetical protein	1.47
**AAEL018075**	hypothetical protein	1.46
**AAEL006989**	cytochrome P450 (CYP6AG7)	1.46
**AAEL005867**	conserved hypothetical protein	1.46
**AAEL004615**	hypothetical protein	1.45
**AAEL007024**	cytochrome P450 (CYP6AG3)	1.45
**AAEL001674**	serine-type enodpeptidase, putative	1.45
**AAEL006161**	Clip-Domain Serine Protease family B (CLIPB31)	1.45
**AAEL007606**	hypothetical protein	1.44
**AAEL005332**	hypothetical protein	1.44
**AAEL015277**	conserved hypothetical protein	1.43
**AAEL015520**	protein-l-isoaspartate o-methyltransferase	1.43
**AAEL003609**	neurobeachin	1.43
**AAEL010435**	conserved hypothetical protein	1.42
**AAEL004447**	hypothetical protein	1.42
**AAEL001621**	conserved hypothetical protein (allergen domain)	1.42
**AAEL005782**	conserved hypothetical protein	1.42
**AAEL008780**	serine-type enodpeptidase, putative	1.42
**AAEL014734**	saccharopine dehydrogenase	1.42
**AAEL000825**	eukaryotic peptide chain release factor GTP-binding subunit (erf2)	1.42
**AAEL013584**	conserved hypothetical protein	1.41
**AAEL011224**	hypothetical protein	1.41
**AAEL001580**	otefin, putative	1.41
**AAEL007591**	conserved hypothetical protein	1.41
**AAEL001938**	ATP-binding cassette sub-family A member 3, putative	1.41
**AAEL001594**	conserved hypothetical protein	1.41
**AAEL011314**	epoxide hydrolase	1.41
**AAEL017284**	Hypothetical protein	1.41
**AAEL002268**	hypothetical protein	1.40

Table 1E: ZIKV and DENV shared downregulated

Column ID	Description	ZIKV FC	DENV FC
**AAEL017835**	18S rRNA	-5.18	-3.38
**AAEL012644**	conserved hypothetical protein	-2.68	-2.77
**AAEL008106**	hypothetical protein	-2.27	-1.72
**AAEL014128**	hypothetical protein	-2.21	-2.65
**AAEL017345**	Hypothetical protein	-2.20	-1.54
**AAEL007703**	conserved hypothetical protein	-2.15	-2.06
**AAEL016864**	tRNA-Ala	-2.03	-2.32
**AAEL002046**	cytochrome P450 (CYP6CB1)	-1.91	-2.09
**AAEL015631**	asparagine synthetase	-1.86	-1.57
**AAEL009018**	cytochrome P450	-1.84	-1.99
**AAEL016423**	tRNA-Ala	-1.72	-2.46
**AAEL000931**	alkaline phosphatase	-1.70	-1.63
**AAEL001098**	clip-domain serine protease, putative	-1.61	-1.55
**AAEL002889**	hypothetical protein	-1.59	-1.93
**AAEL004022**	carboxylesterase	-1.58	-1.76
**AAEL013812**	hypothetical protein (chitin-binding domain type 2)	-1.56	-2.00
**AAEL009181**	conserved hypothetical protein	-1.55	-2.73
**AAEL011763**	prophenoloxidase (PPO3)	-1.51	-1.49
**AAEL006834**	glutamate semialdehyde dehydrogenase	-1.50	-1.51
**AAEL015045**	transcription factor IIIA, putative	-1.48	-1.49
**AAEL012871**	hypothetical protein	-1.48	-1.50
**AAEL007738**	app binding protein	-1.45	-1.57
**AAEL006594**	serine-type enodpeptidase, putative	-1.43	-1.57

Table 1F: ZIKV and DENV shared upregulated

Column ID	Description	ZIKV FC	DENV FC
AAEL005702	conserved hypothetical protein	7.05	2.02
AAEL013350	heat shock protein 26kD, putative	6.80	8.32
AAEL013349	lethal(2)essential for life protein, l2efl	5.76	4.96
AAEL013118	conserved hypothetical protein	5.34	4.06
AAEL013345	alphaA-crystallin, putative	5.30	4.70
AAEL017975	heat shock protein HSP70 (HSP70Ba)	4.57	3.08
AAEL017931	U1 spliceosomal RNA	3.22	2.86
AAEL003849	defensin anti-microbial peptide (DEFE)	3.20	2.46
AAEL018663	tRNA-Leu	2.97	5.37
AAEL013352	lethal(2)essential for life protein, l2efl	2.81	3.28
AAEL004157	hypothetical protein	2.69	3.01
AAEL017757	U5 spliceosomal RNA	2.54	3.01
AAEL000611	cecropin anti-microbial peptide (CECE)	2.45	2.26
AAEL000668	conserved hypothetical protein	2.42	2.41
AAEL006305	conserved hypothetical protein	2.39	1.63
AAEL017514	hypothetical protein	2.37	2.11
AAEL017976	heat shock protein HSP70 (HSP70Bb)	2.32	2.66
AAEL013346	lethal(2)essential for life protein, l2efl	2.28	1.99
AAEL004317	hypothetical protein	2.28	1.98
AAEL007780	conserved hypothetical protein	2.26	1.91
AAEL010137	ketoreductase, putative	2.19	3.03
AAEL007913	sulfotransferase (sult)	2.16	3.11
AAEL001905	conserved hypothetical protein	2.15	1.98
AAEL008769	serine-type enodpeptidase, putative	2.03	1.54
AAEL000586	hypothetical protein	2.03	1.67
AAEL017292	Hypothetical protein	2.02	1.80
AAEL001833	Juvenile hormone-inducible protein, putative	1.95	1.50
AAEL017144	Hypothetical protein	1.95	1.66
AAEL010670	lethal(2)essential for life protein, l2efl	1.92	2.17
AAEL005986	hypothetical protein	1.91	1.65
AAEL006723	conserved hypothetical protein	1.90	1.67
AAEL015053	conserved hypothetical protein	1.90	1.77
AAEL000607	hypothetical protein	1.89	2.06
AAEL014921	lipase 1 precursor	1.86	2.02
AAEL017231	hypothetical protein	1.81	3.55
AAEL003589	transcription factor, putative	1.78	1.64
AAEL017826	U1 spliceosomal RNA	1.74	1.51
AAEL017413	Hypothetical protein	1.65	1.65
AAEL018684	NADH dehydrogenase subunit 6 (ND6)	1.64	1.63
AAEL009058	hypothetical protein	1.59	1.85
AAEL007004	GPCR Bride of Sevenless Family (GPRBOS1)	1.55	1.97
AAEL013699	hypothetical protein	1.54	1.56
AAEL010547	hypothetical protein	1.54	2.14
AAEL012284	DNA-directed RNA polymerase I 16 kDa polypeptide	1.52	2.28
AAEL017851	Small nucleolar RNA U3	1.51	1.64
AAEL018672	tRNA-Arg	1.50	2.03
AAEL011110	conserved hypothetical protein	1.49	1.53
AAEL001054	glutathione S-transferase (GSTD4)	1.49	2.31
AAEL001087	synaptic vesicle protein	1.48	2.05

AAEL002603	triacylglycerol lipase, putative	1.47	1.98
AAEL003841	defensin anti-microbial peptide (DEFA)	1.46	2.00
AAEL012895	meiotic coiled-coil protein, putative	1.46	1.59
AAEL005849	synaptic vesicle protein	1.45	1.51
AAEL018691	ssu rRNA	1.45	1.59
AAEL007262	hypothetical protein	1.42	1.55
AAEL017646	U1 spliceosomal RNA	1.41	1.69
