# Supplementary material for: Aedes aegypti Molecular Responses to Zika Virus: Modulation of Infection by the Toll and Jak/Stat Immune Pathways and Virus Host Factors
Source: Front Microbiol. 2017 Oct 23;8:2050. doi: 10.3389/fmicb.2017.02050 (PMC5660061; doi:10.3389/fmicb.2017.02050)
Supplement: Supplementary file 4 [file Table_2.PDF]

Table S2: Primer sequences used for dsRNA synthesis and qRT-PCR. Sequences in bold corresponds to T7 promoter.

Primer Name	Sequence (5' to 3')
dsGFP F	**TAATACGACTCACTATAGGG**ATGGTGAGCAAGGGCGAGGAGCTGT
dsGFP R	**TAATACGACTCACTATAGGG**TTACTTGTACAGCTCGTCCATGCCG
ds Cactus F	**TAATACGACTCACTATAGGG**CGAGTCAACAGAACCCGAGCAG
ds Cactus R	**TAATACGACTCACTATAGGG**TGGCCCGTCAGCACCGAAAG
ds Caspar F	**TAATACGACTCACTATAGGG**GGAAGCAGATCGAGCCAAGCAG
ds Caspar R	**TAATACGACTCACTATAGGG**GCATTGAGCCGCCTGGTGTC
ds PIAS F	**TAATACGACTCACTATAGGG**GATACACCCAGCAAGCCAGT
ds PIAS R	**TAATACGACTCACTATAGGG**AACCCCGCTATTGTATGCTG
ds vATP-VoB F	**TAATACGACTCACTATAGGG**CGGTTACTCCTTCACTGGCA
ds vATP-VoB R	**TAATACGACTCACTATAGGG**CGTAAATGGCAACAGCCTCG
ds vATP-ac39 F	**TAATACGACTCACTATAGGG**AAGTGCGGAATCCTCAAGCA
ds vATP-ac39 R	**TAATACGACTCACTATAGGG**ATGCAGTCGACGAAGAAGGG
ds IMPDH F	**TAATACGACTCACTATAGGG**GGAAGAAGATGTCGCCCTTA
ds IMPDH R	**TAATACGACTCACTATAGGG**CTCTTGCGTAATGCAGATGG

Primer Name	Sequence (5' to 3')
RT S7 F	GGGACAAATCGGCCAGGCTATC
RT S7 R	TCGTGGACGCTTCTGCTTGTTG
RT Cactus F	AGACAGCCGCACCTTCGATTCC
RT Cactus R	CGCTTCGGTAGCCTCGTGGATC
RT Caspar F	GAATCCGAGCGAGCCGATGC
RT Caspar R	CGTAGTCCAGCGTTGTGAGGTC
RT PIAS F	GCTGCAACGCATGAAAACTA
RT PIAS R	CAGACGGGACAGTTCCAAGT
RT vATP-V0B F	TCGGTTATCTTCTGCGAGGC
RT vATP-V0B R	CCGACAGCGATACCACAGAA
RT vATP-ac39 F	GTACCTGGAGGCGTTCTACG
RT vATP-ac39 R	GGGATACAACTTGGCACGGT
RT IMPDH F	CCTCAAGCCACTGCTGTGTA
RT IMPDH R	ATGCTCCCAAGGAAAGAGCC
