# Supplementary material for: Aedes aegypti Molecular Responses to Zika Virus: Modulation of Infection by the Toll and Jak/Stat Immune Pathways and Virus Host Factors
Source: Front Microbiol. 2017 Oct 23;8:2050. doi: 10.3389/fmicb.2017.02050 (PMC5660061; doi:10.3389/fmicb.2017.02050)
Supplement: Supplementary file 5 [file Table_3.PDF]

Table S3. **Descriptive statistics of ZIKV and DENV infected mosquitoes**

	**Fig.1A- Orl Midgut**				**Fig.1B - Rock Midgut**			
	Orl				Rock			
	4d	7d	10d	14d	4d	7d	10d	14d
N	34	36	46	36	37	40	42	42
Mean	4462	26439	13962	11308	28288	51163	31762	10107
Median	375	11000	4500	3000	8500	37500	32500	5000
SD	10108	34464	23082	29139	34924	46346	25457	19821

	**Fig.1C- Midgut Strain comparison**							
	Orl				Rock			
	4d	7d	10d	14d	4d	7d	10d	14d
N	3	3	3	3	3	3	3	3
Mean	1125	16250	7750	2833	23450	45000	31083	5083
Median	1375	19250	4000	2500	8500	40000	35000	4750
SEM	591	6105	5865	333	18376	7638	6577	464

P value	4d vs. 7d	0.0012
	4d vs. 10d	0.0786
	4d vs. 14d	0.5414
	7d vs. 10d	0.8437
	7d vs. 14d	0.2406
	10d vs. 14d	> 0.9999

4d vs. 7d	0.009
4d vs. 10d	0.4687
4d vs. 14d	0.1386
7d vs. 10d	0.833
7d vs. 14d	< 0.0001
10d vs. 14d	0.0002

P value	4d Orl vs. Rock	< 0.0001
	7d Orl vs. Rock	0.0019
	10d Orl vs. Rock	< 0.0001
	14d Orl vs. Rock	0.1103

	**Fig.1D- Orl Abdomen**				**Fig.1E- Rock Abdomen**			
	Orl				Rock			
	4d	7d	10d	14d	4d	7d	10d	14d
N	35	38	25	51	56	61	40	55
Mean	40	38	193	3544	8	154	498	8219
Median	0	0	0	45	0	0	70	850
SD	197	133	720	12363	53	963	1159	28855

	**Fig.1F- Abdomen Strain comparison**							
	Orl				Rock			
	4d	7d	10d	14d	4d	7d	10d	14d
N	3	3	3	3	3	3	3	3
Mean	0	0	2	58	0	0	48	738
Median	0	0	0	30	0	0	45	900
SEM	0	0	2	44	0	0	28	268

P value	4d vs. 7d	> 0.9999
	4d vs. 10d	0.1292
	4d vs. 14d	< 0.0001
	7d vs. 10d	0.5970
	7d vs. 14d	< 0.0001
	10d vs. 14d	0.0644

4d vs. 7d	0.2306
4d vs. 10d	< 0.0001
4d vs. 14d	< 0.0001
7d vs. 10d	0.0007
7d vs. 14d	< 0.0001
10d vs. 14d	0.0795

P value	4d Orl vs. Rock	0.4013
	7d Orl vs. Rock	0.0261
	10d Orl vs. Rock	0.0013
	14d Orl vs. Rock	0.0009

	**Fig 1G- Orl Salivary glands**			**Fig 1H- Orl Salivary glands**		
	Orl			Rock		
	10d	14d	21d	10d	14d	21d
N	33	38	27	40	46	34
Mean	114	3606	17333	0	70	263
Median	0	0	50	6797	4650	27271
SD	436	15500	36135	33742	10714	51991

	**Fig 1I- Salivary glands Strain comparison**					
	Orl			Rock		
	10d	14d	21d	10d	14d	21d
N	3	3	3	3	3	3
Mean	0	57	1022	7	335	5837
Median	0	15	30	0	23	8500
SEM	0	49	1002	7	320	2917

P value	10d vs. 14d	0.0361
	10d vs. 21d	0.0002
	14d vs. 21d	0.2497

P value	10d Orl vs. Rock	0.3288
	14d Orl vs. Rock	0.0650
	21d Orl vs. Rock	0.5106

Fig 2B-C RNAseq statistics summary

Sample Name	Total Reads	Counted Fragments	% of Total
Control Rep 1	42,752,949	36,262,526	84.82
Control Rep 2	44,153,788	37,277,759	84.43
Control Rep 3	48,917,412	40,865,661	83.54
DENV Rep 1	45,078,409	37,332,840	82.82
DENV Rep 2	45,381,694	38,111,764	83.98
DENV Rep 3	49,181,998	42,014,600	85.43
ZIKV Rep 1	45,682,152	37,669,587	82.46
ZIKV Rep 2	36,533,643	30,364,072	83.11
ZIKV Rep 3	42,430,392	35,518,615	83.71

	**Fig.2A- RNAseq**		**Fig.3E- Immune pathways**				**Fig.4A- Host factors**			
	DENV	ZIKV	Control	Toll	Imd	Jak/Stat	Control	VoB	Ac39	IMPDH
N	20	20	62	65	66	65	65	41	65	63
Mean	4409	26782	52088	21086	42576	26721	20987	5038	6734	12522
Median	3000	900	35000	6500	27500	11500	5000	1000	1000	800
SD	4143	53157	56045	31625	44562	33126	34330	9962	17035	22853
P value	-	0.315	-	0.0003	0.7348	0.022	-	0.0266	0.006	0.0431
